# Epigallocatechin Gallate Relieved PM2.5-Induced Lung Fibrosis by Inhibiting Oxidative Damage and Epithelial-Mesenchymal Transition through AKT/mTOR Pathway

**DOI:** 10.1155/2022/7291774

**Published:** 2022-06-06

**Authors:** Zhou Zhongyin, Wang Wei, Xiong Juan, Fan Guohua

**Affiliations:** ^1^Department of Gastroenterology, Renmin Hospital of Wuhan University, Wuhan 430060, China; ^2^Department of Thoracic Surgery, Renmin Hospital of Wuhan University, Wuhan 430060, China

## Abstract

Oxidative damage and epithelial-mesenchymal transition (EMT) are main pathological processes leading to the development of PM2.5-induced lung fibrosis. Epigallocatechin gallate (EG), a natural polyphenol extracted from green tea, possesses the ability to combat oxidative stress and inflammation. However, the potential roles of EG in PM2.5-induced lung fibrosis have not been reported yet. In the present study, we investigated whether EG could relieve PM2.5-induced lung injury and fibrosis *in vivo* and *in vitro*. To mimic PM2.5-induced lung fibrosis, C57/BL6 mice were intranasally instilled with PM2.5 suspension, and MLE-12 lung epithelial cells were stimulated with PM2.5 (100 *μ*g/mL) *in vitro*. The results showed that intragastric administration of EG (20 mg/kg/d or 80 mg/kg/d for 8 weeks) significantly prevented lung injury, inflammation, and oxidative stress in PM2.5-induced mice, apart from inhibiting collagen deposition. Additionally, EG treatment also suppressed the activation of AKT/mTOR signaling pathway in lung tissues challenged with PM2.5. *In vitro* experiments further demonstrated that EG treatment could enhance cell viability in a concentration-dependent manner in PM2.5-treated MLE-12 lung epithelial cells. Also, the overexpression of constitutively active AKT could offset the inhibitory effects of EG on EMT and oxidative stress in PM2.5-treated MLE-12 lung epithelial cells. Finally, AKT overexpression also blocked the inhibitory effect of EG on the phosphorylation of mTOR in PM2.5-treated MLE-12 lung epithelial cells. In conclusion, EG could improve PM2.5-induced lung fibrosis by decreasing oxidative damage and EMT through AKT/mTOR pathway, which might be a potential candidate for the treatment of PM2.5-induced lung fibrosis.

## 1. Introduction

Air pollution has posed huge threat to human health, especially the cardiovascular system and respiratory system [1]. PM2.5 is defined as ambient air particulate matter with aerodynamic diameters less than 2.5 *μ*m, which is one of the important pollutant compositions in air [2]. Increased PM2.5 is implicated with a variety of chronic diseases including bronchitis, chronic obstructive pulmonary disease (COPD) [3], asthma [4], coronary artery disease [5], and atherosclerosis [6]. PM2.5 can be easily inhaled into the airway and subsequently deposited in lung alveolar space due to its small size, the long-term deposition of which further gives rise to lung pathological injury and fibrosis by increasing oxidative stress and epithelial-mesenchymal transition (EMT) [7]. Thereby, candidates with antioxidative and antifibrotic effects possess the potential to attenuate PM2.5-induced lung injury and fibrosis.

At present, the pathogenesis of lung fibrosis has not been well clarified. Lung fibrosis originates from aberrant repair of the lung epithelial cells and repeated injury [8]. Some studies hold the view that the progressive pulmonary dysfunction declining resulted from the lung epithelial injury as well as aberrant fibroblast proliferation participated in the development of inflammation and lung fibrosis [9, 10]. Additionally, EMT and excessive extracellular matrix (ECM) production also result in pulmonary structural remodeling [11, 12]. In recent years, a great many researchers have been immersed in the roles of lung epithelial cells and EMT in fibrosis. Lung epithelial cells could differentiate into a mesenchymal phenotype through EMT, which further secrete a series of fibrogenic cytokines to activate fibroblasts [13]. Protein kinase B (PKB, also known as AKT)/mechanistic target of rapamycin (mTOR) pathway serves as one of the most important pathways contributing to the activation of EMT, playing an essential role in the progression of lung fibrosis [14]. Hence, blocking AKT/mTOR pathway has been proposed to be a promising strategy to suppress EMT and combat lung fibrosis.

Epigallocatechin gallate (EG) is considered as the most abundant polyphenol with bioactivity in green tea isolated from *Camellia sinensis* [15]. As a bioactive dietary component, EG owns a great many pharmacological actions including antioxidation, anti-inflammation, antifibrosis, and inhibiting endoplasmic reticulum stress [16–18]. In acute lung injury induced by sepsis, acute pancreatitis, paraquat, and hip fracture, EG treatment could exert vital protective roles by different mechanisms [19–22]. In transverse aortic constriction-induced cardiac fibrosis, EG treatment significantly decreased collagen deposition and cardiomyocyte hypertrophy by blocking AKT/mTOR pathway [18]. These facts raised the possibility that EG may own the potential of pulmonary protection. Yet whether the EG could protect against chronic lung injury is still unknown.

In the current study, we aimed to explore whether EG can attenuate lung injury and fibrosis induced by PM2.5in mice and to clarify the underlying mechanisms.

## 2. Materials and Methods

### 2.1. Regents and Chemicals

Epigallocatechin gallate with a purity of 99.87% was purchased from MedChemExpress Co., Ltd. (#: HY-13653, Shanghai, China). Primary antibodies against phosphorylated (P)-AKT, total (T)-AKT, P-mTOR, T-mTOR, and GAPDH were provided by Abcam (Cambridge, UK), while secondary antibody was obtained from LI-COR Biosciences (Lincoln, USA). Trypsin-EDTA (0.25%) phenol red and Dulbecco's modified Eagle's medium nutrient mixture F-12 (DMEM/F-12), fetal bovine serum (FBS), and trypsin-ethylenediaminetetraacetic acid (0.25%) phenol were provided by Invitrogen-Gibco (Grand Island, NY, USA).

### 2.2. The Extraction of PM2.5 Sampling

PM2.5 samples were obtained with a high flow PM2.5 sampler (Ecotech, Australia) in the Wuhan Environment Surveillance Centre. The extraction and analysis of PM2.5 sampling were carried out based on our previous study [23]. The PM2.5 particles were stored at −20°C and detached from filters by sonication, which were next desiccated by lyophilization. At last, these PM2.5 solid particles were suspended in phosphate buffer saline (PBS) evenly by vortex concussion for intranasal instillation and cell experiments.

### 2.3. Mice and Models

C57/BL6J male mice weighing 24.3 ± 1.7 g (8~10 weeks) were provided by the Chinese Academy of Medical Sciences (Beijing). Mice were housed in a specific pathogen-free rooms with constant temperature (22–25°C) and humidity (50–60%) under a 12 h light/dark cycle. Mice had ad libitum access to food and water. All animal experimental procedures were in accordance with the Guidelines for the Care and Use of Laboratory Animals published by the National Institutes of Health and were approved by the Committee on the Laboratory Animal Welfare & Ethics of Renmin Hospital of Wuhan University.

According to previous study, mice were intranasally instilled with PM2.5 particulates (100 mg/kg bodyweight) suspended in 50 *μ*L of sterile water or isovolumetric sterile saline once a week for four weeks to induce PM2.5-induced lung injury and fibrosis [24]. Meanwhile, EG (20 mg/kg/d or 80 mg/kg/d) or equal volume of sterile saline was administered orally to mice for continuous 8 weeks. 56 days after EG administration, mice were sacrificed by cervical dislocation after they were anaesthetized deeply by isoflurane inhalation. And the lungs were excised for molecular biological and histological detections. To keep the lungs in an extended status, 4% paraformaldehyde was instilled in the trachea.

### 2.4. Histopathological Analysis

The left lung tissues were embedded in paraffin, which were then sliced into 3 *μ*m-thick sections to expose the cross section of lung tissue. Subsequently, the sections were stained using hematoxylin for a few minutes and rinsed by tap water, followed by the differentiation with 1% hydrochloric acid alcohol for 3-5 seconds. After that, the sections were stained with eosin for 2-3 minutes. The lung injury was then observed and scored under a microscope. As described previously, a semiquantitative scoring system was applied to access lung injury based on the degree of neutrophil infiltration and pulmonary edema. Each index was scored by a pathologist according to the following criteria: 0, no injury; 1, injury up to 25% of the field; 2, injury up to 50% of the field; 3, injury up to 75% of the field; and 4, diffuse injury. A total lung injury score was calculated by summing up the two components (neutrophil infiltration and pulmonary edema) [25].

As for Masson staining, the lung sections were stained with iron hematoxylin for the nucleus after dewaxed in water. After that, the sections were stained with ponceau red, followed by phosphomolybdic acid aqueous solution. Next, the sections were stained with aniline blue and sealed with neutral resin. At last, the sections were observed and pictured under a light microscope.

### 2.5. Cell Culture and Treatment

The MLE-12 lung epithelial cells were provided by Kunming Cell Bank of Typical Cultures Preservation Committee, Chinese Academy of Sciences (Kunming, China). The cells were cultured in DMEM/F-12 supplemented with FBS (10%) at 37°C in an incubator with 5% CO_2_. To establish an *in vitro* model of PM2.5-induced lung injury, the MLE-12 lung epithelial cells were stimulated with PM2.5 (100 *μ*g/mL) with or without EG (20 *μ*M) for 24 hours as described [23]. To activate AKT in MLE-12 lung epithelial cells constitutively, the adenoviral (Ad) vectors generated by Hanbio Biotechnology Co. (Shanghai, China) were used in the present study [26].

### 2.6. Western Blot and Quantitative Real-Time RT-PCR

The Western blot was carried out to access the levels of protein expression. The total proteins in lung tissues and lung epithelial cells were extracted using RIPA lysis buffer containing a protease inhibitor cocktail. A bicinchoninic acid (BCA) protein assay kit was used to detect and normalize the protein concentration. 10 *μ*L of markers or quantitative protein samples (50 *μ*g) was added into a 10% SDS-PAGE and separated before being transferred to the polyvinylidene fluoride (PVDF) membranes. Subsequently, 5% skim milk was used to block the nonspecific protein binding sites in the PVDF membranes. Then, the membranes were incubated with antibodies against GAPDH (1: 1000), P-mTOR (1 : 500), T-mTOR (1 : 1000), P-AKT (1 : 5000), and T-AKT (1 : 1000) diluted in Tris-buffered saline+Tween (TBST) overnight at 4°C overnight, respectively. The next day, the PVDF membranes were incubated with the secondary antibodies after washed with TBST for 3 times. ECL detection kits were applied to access chemiluminescence using a LI-COR Odyssey image system. The protein expression levels were normalized to GAPDH as an internal reference. The expression was quantified using the Image Lab software from Bio-Rad (Hercules, CA, USA).

Total RNA was extracted from murine lung tissues and cultured lung epithelial cells using the TRIzol reagent. Next, total RNA was reversely transcribed to complementary DNA by oligo primers using the Maxima First Strand cDNA Synthesis Kit. The transcriptional levels of target genes including TNF-*α*, IL-1*β*, MCP-1, collagen I, collagen III, connective tissue growth factor (CTGF), E-Cadherin, *α*-SMA, Snail, thioredoxin-interacting protein (TXNIP), thioredoxin reductase 1(TXNRD1), and thioredoxin 1 (TXN1) were detected with SYBR green. GAPDH was used as an internal reference gene to calculate the relative expression of target genes.

### 2.7. The Determination of Oxidative Stress

The fresh murine lung tissues (100 mg) were homogenized and then centrifuged for 10 min (X4230 g) to obtain the supernatant fractions. Then, the NADH oxidase (NOX) and superoxide dismutase (SOD) activities and malondialdehyde (MDA) level were detected using the commercially available kits according to the instructions [27].

### 2.8. CCK8 Assay

Cell Counting Kit-8 (CCK8) assay was performed to detect cell viability using aCCK8 assay kit. In detail, the lung epithelial cells were seeded into 96-well plates. Subsequently, 10 *μ*L of CCK8 working solution was added into each well in a dark environment. Then, the plates were kept in an incubator (5% CO_2_) at 37°C for 1 hour. Finally, the absorbance was measured at 450 nm to evaluate the cell viability of lung epithelial cells.

### 2.9. Data Analysis

All data in this study are presented as the mean ± standard deviation (SD). One-way ANOVA followed by a post hoc Tukey's test was performed to analyze the differences among multiple groups, while an unpaired, two-sided Student *t* test was carried out to analyze the differences between 2 groups. *P* < 0.05 was defined as statistical significance.

## 3. Results

### 3.1. EG Treatment Decreased Pathological Injury and Inflammatory Response in Lung Tissues Treated with PM2.5

**Figure 1:**
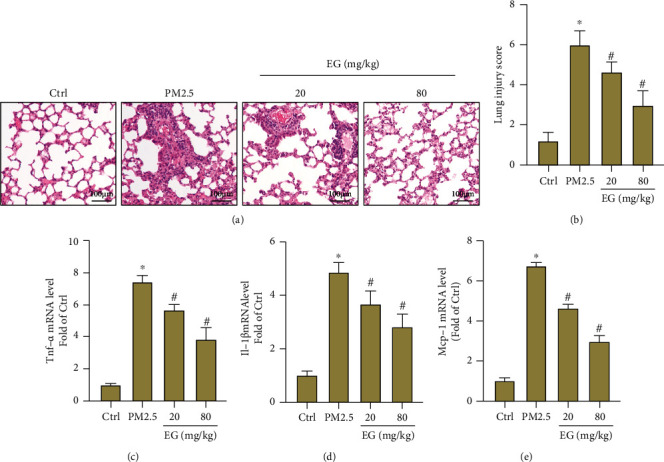
EG treatment decreased pathological injury and inflammatory response in lung tissues treated with PM2.5. (a–b) H&E staining of lung tissues in the indicated groups and lung injury scores (*n* = 5). Original magnification ×200. (c–e) Comparison of mRNA levels of TNF-*α*, IL-1*β*, and MCP-1 in lung tissues (*n* = 5).∗*P* < 0.05 vs. Ctrl group, #*P* < 0.05 vs. PM2.5 group.

To begin with, lung pathological injury in each group was assessed by H&E staining. The results showed that EG (both 20 mg/kg/d and 80 mg/kg/d) could significantly alleviate interstitial thickening and inflammatory cell infiltration caused by PM2.5, which was reflected by decreased lung injury score (Figures 1(a) and 1(b)). The results from RT-PCR also indicated that EG treatment significantly decreased inflammatory response in PM2.5-induced lung tissues, which was evidenced by decreased mRNA levels of TNF-*α*, IL-1*β*, and MCP-1(Figures 1(c)–1(e)).

### 3.2. EG Treatment Inhibited Oxidative Stress in Lung Tissues Treated with PM2.5

**Figure 2:**
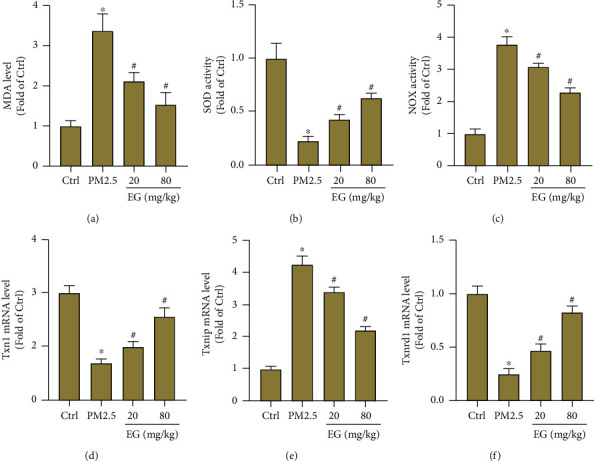
EG treatment inhibited oxidative stress in lung tissues treated with PM2.5. (a) MDA level in lung tissues (*n* = 5). (b–c) SOD activity and NOX activity in lung tissues (*n* = 5). (d–f) Comparison of mRNA levels of Txn1, Txnip, and Txnrd1 in lung tissues (*n* = 5). ∗*P* < 0.05 vs. Ctrl group, #*P* < 0.05 vs. PM2.5 group.

Next, the levels of oxidative stress were evaluated using different methods. As shown in Figure 2(a), the level of lipid peroxidative product MDA in lung tissues from mice challenged PM2.5 was suppressed after EG (both 20 mg/kg/d and 80 mg/kg/d) treatment. Also, the activity of SOD and NOX was determined in each group. The results demonstrated that EG treatment could increase SOD activity and decreased NOX activity in PM2.5-induced lung tissues, indicating that EG exerted antioxidant effects (Figures 2(b) and 2(c)). Meanwhile, we observed the effects of EG on the mRNA levels of pro-oxidant gene TXNIP and antioxidant genes including TXN1 and TXNRD1. The results showed that EG treatment upregulated the mRNA levels of TXN1 and TXNRD1 and downregulated the mRNA level of TXNIP (Figures 2(d)–2(f)).

### 3.3. EG Alleviated Fibrosis and EMT in PM2.5-Induced Lung Tissues

**Figure 3:**
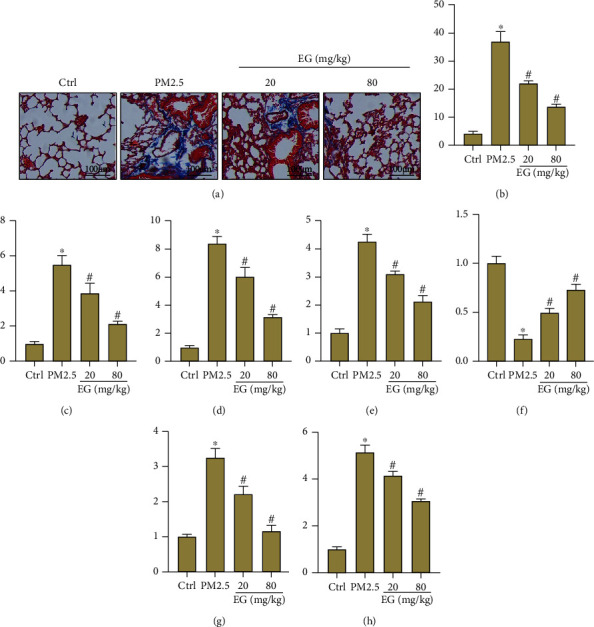
EG alleviated fibrosis and EMT in PM2.5-induced lung tissues. (a–b) Masson staining of lung tissues in the indicated groups and the quantification of fibrosis (*n* = 5). Original magnification ×200. (c–e) Comparison of mRNA levels of fibrosis markers including collagen I, collagen III, and CTGF in lung tissues (*n* = 5). (f–h) Comparison of mRNA levels of EMT markers including E-Cadherin, *α*-SMA, and Snail in lung tissues (*n* = 5). ∗*P* < 0.05 vs. Ctrl group, #*P* < 0.05 vs. PM2.5 group.

Fibrosis is one of the most important features of PM2.5-induced lung injury [28]. Hence, we also observed the effects of EG on lung fibrosis of mice challenged with PM2.5. As shown in Figures 3(a) and 3(b), EG (both 20 mg/kg/d and 80 mg/kg/d) treatment significantly decreases the fibrotic area in lung tissues from mice treated with PM2.5. Furthermore, the mRNA levels of collagen I, collagen III, and CTGF in PM2.5-induced lung tissues were also inhibited by EG (Figures 3(c)–3(e)). Considering that EMT serves as one vital mechanism contributing to lung fibrosis, we also detected the markers of EMT in lung tissues. The results (Figures 3(f)–3(h)) showed that EG treatment significantly increased the level of E-Cadherin and decreased the levels of *α*-SMA and Snail, suggesting that EG shifted the phenotype from mesenchymal cells to epithelial cells.

### 3.4. AKT/mTOR Pathway Was Inhibited by EG in Lung Tissues Treated with PM2.5

**Figure 4:**
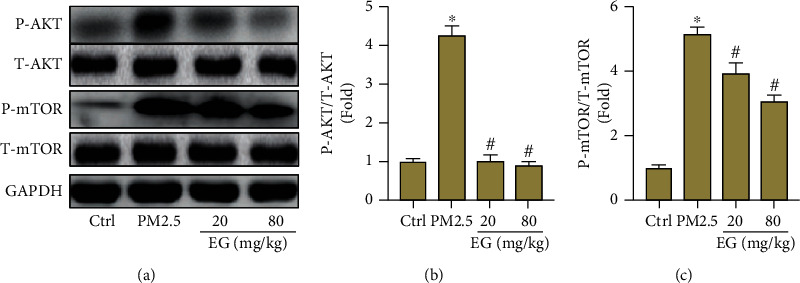
AKT/mTOR pathway was inhibited by EG in lung tissues treated with PM2.5. (a–c) Western blot analysis and statistical results for P-AKT, T-AKT, P-mTOR, and T-mTOR (*n* = 5).∗*P* < 0.05 vs. Ctrl group, #*P* < 0.05 vs. PM2.5 group.

Previous studies have illustrated that the abnormal activation of AKT/mTOR pathway was implicated with oxidative stress and EMT [29, 30]. Here, our data disclosed that PM2.5 stimulation significantly promoted the phosphorylation of AKT and mTOR in lung tissues (Figures 4(a)–4(c)). As expected, EG (both 20 mg/kg/d and 80 mg/kg/d) treatment obviously blocked the activation of AKT/mTOR pathway, hinting that the pulmonary protection from EG may be associated with the inhibition of AKT/mTOR pathway.

### 3.5. EG Relieved EMT and Oxidative Stress in PM2.5-Induced Lung Epithelial Cells in an AKT-Dependent Manner

**Figure 5:**
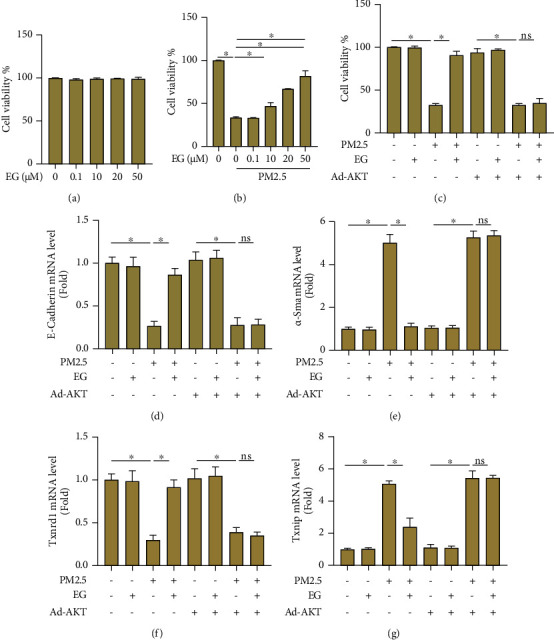
EG relieved EMT and oxidative stress in PM2.5-induced lung epithelial cells in an AKT-dependent manner. (a) Cell viability of lung epithelial cells treated with different concentrations of EG (*n* = 5). (b) Cell viability of PM2.5-induced lung epithelial cells treated with different concentrations of EG (*n* = 5). (c). Cell viability of PM2.5-induced lung epithelial cells treated with EG (20 *μ*M) after AKT was overexpressed by adenovirus infection (*n* = 5). (d–e) Comparison of mRNA levels of EMT markers including E-Cadherin and *α*-SMA in the indicated groups (*n* = 5). ∗*P* < 0.05 vs. the indicated groups; ns: no significance.

Next, we investigated the roles of EG in PM2.5-induced lung epithelial cells. First, cell viability was detected in lung epithelial cells treated with different concentrations of EG. As shown in Figure 5(a), the concentrations ranging from 0.1 to 50 *μ*M display no effects on cell viability of lung epithelial cells. In PM2.5-induced lung epithelial cells, the concentrations ranging from 10 to 50 *μ*M improved cell viability (Figure 5(b)). Based on these data, 20 *μ*M was selected for the subsequent *in vitro* experiments. The results showed that EG (20 *μ*M) could improve cell viability of PM2.5-induced lung epithelial cells, whereas constitutively active AKT overexpression by adenoviral infection blocked the effects of EG on cell viability (Figure 5(c)). Similarly, AKT activation also offset the inhibitory effects of EG on EMT in PM2.5-induced lung epithelial cells, evidenced by decreased mRNA level of E-Cadherin and increased mRNA level of *α*-SMA (Figures 5(d) and 5(e)). Additionally, the antioxidant effect of EG on PM2.5-induced lung epithelial cells could also be counteracted after AKT activation (Figures 5(f) and 5(g)). Taken together, these results disclosed the fact that EG could inhibit EMT and oxidative stress in PM2.5-induced lung epithelial cells in an AKT-dependent manner.

### 3.6. AKT/mTOR Pathway Was Involved in Pulmonary Protection from EG in PM2.5-Induced Lung Epithelial Cells

**Figure 6:**
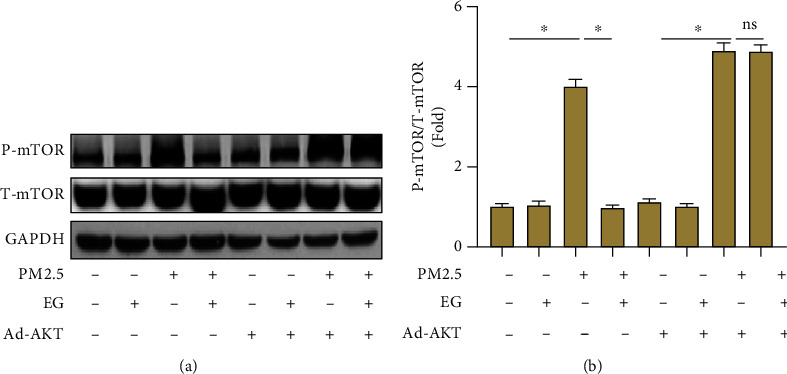
AKT/mTOR pathway was involved in pulmonary protection from EG in PM2.5-induced lung epithelial cells. (a–b) Western blot analysis and statistical results for P-mTOR and T-mTOR (*n* = 5). ∗*P* < 0.05 vs. the indicated groups; ns: no significance.

Finally, we detected the protein levels of P-mTOR and T-mTOR in the indicated groups. As shown in Figures 6(a) and 6(b), EG could not inhibit mTOR at baseline, whereas it significantly decreased the protein level of P-mTOR inPM2.5-induced lung epithelial cells. As expected, AKT activation offset the inhibitory effects of EG on mTOR, which further solid the hypothesis that EG exerted its protective effects by blocking the activation of AKT/mTOR pathway in PM2.5-induced lung injury and fibrosis.

## 4. Discussion

**Figure 7:**
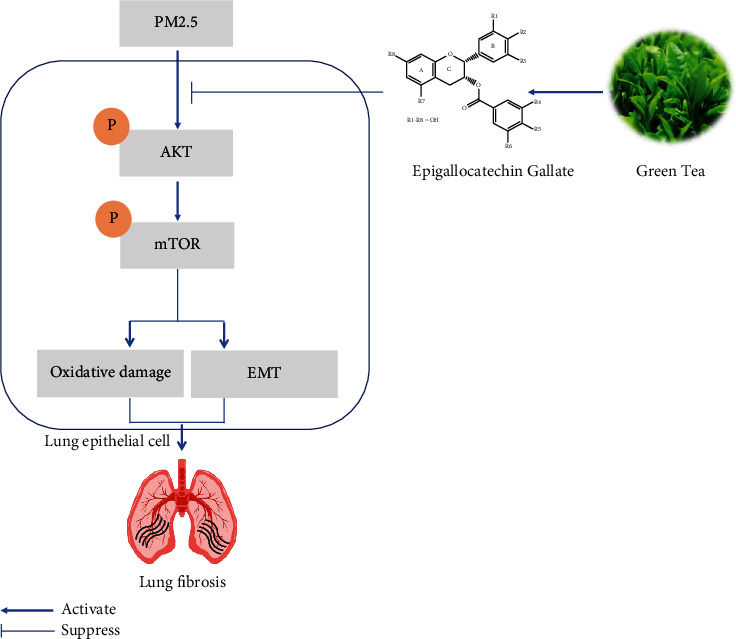
The possible mechanisms by which EG relieves PM2.5-induced lung fibrosis. PM2.5 could increase the phosphorylation of AKT, which subsequently phosphorylated mTOR in lung epithelial cell. The activated AKT/mTOR pathway further gave rise to oxidative damage and EMT, eventually causing lung fibrosis. The natural epigallocatechin gallate extracted from green tea could block the activation of AKT/mTOR, thus improving PM2.5-induced lung fibrosis by decreasing oxidative damage and EMT.

Here, we disclosed that EG treatment prevents oxidative damage, inflammation, and lung fibrosis induced by PM2.5 for the first time. EG blocked the EMT of lung epithelial cells induced by PM2.5 *in vitro*. The pulmonary protection from EG was associated with the inactivation of the AKT/mTOR pathway. Constitutive activation of AKT by adenovirus infection in lung epithelial cells could offset EG-elicited protection (Figure 7).

In many epidemiological studies, the relationship between respiratory diseases and ambient airborne fine particulate matter exposure has been well illustrated [31, 32]. To our knowledge, PM2.5 is comprised of a series of particles involving nitrate, black carbon, sulfate, polycyclic aromatic hydrocarbons, metals, and automobile exhaust particles, which could invade distal small airways and alveoli [33]. Excessive accumulation of PM2.5 in lung parenchyma could lead to irreversible lung fibrosis, apart from inducing inflammation and oxidative stress [34]. In the young and the elderly, long-term PM2.5 exposure gives rise to poorer lung function indices, evidenced by reduced forced vital capacity (FVC), and forced expiratory volume in 1 s (FEV1), as well as peak expiratory flow (PEF) [35]. At present, oxidative stress and EMT are regarded as two main factors contributing to lung fibrosis. Some animal experiments also showed that long-term PM2.5 exposure aggravates lung fibrosis by mediating oxidative stress and AKT activation [36]. PM2.5 displays s a potent redox potential by elevating reactive oxygen species (ROS). In preexisting COPD rats stimulated with PM2.5, a significant reduction of some antioxidant factors including nuclear factor erythroid 2-related factor 2 (Nrf2), total superoxide dismutase, and heme oxygenase-1 was observed [37]. These studies hinted that oxidative stress serves as one critical mechanism contributing to PM2.5-induced lung fibrosis and injury. On the other one hand, the interaction between epithelial cells and basal lamina could be altered by PM2.5, reflected by cytoskeletal and extracellular matrix with mesenchymal features [38]. In the present study, PM2.5 exposure promoted the development of EMT and oxidative damage *in vivo* and *in vitro*, which was significantly blocked by EG treatment.

Both AKT and mTOR (known as the mechanistic target of rapamycin) are serine-threonine protein kinases, participating in protein synthesis, metabolism pathways, autophagy, cell proliferation, and cell growth [39]. Activated AKT could phosphorylate mTOR, promoting the conversion of lung epithelial cells to fibroblasts, inducing lung fibrosis [40]. Additionally, the activation of AKT/mTOR pathway also gives rise to oxidative stress, which further aggravated the development of fibrosis [29, 41]. Here, our study unveiled that EG treatment not only decreased oxidative stress but also inhibited EMT during PM2.5-induced lung fibrosis by blocking the activation of AKT/mTOR pathway. In fact, Nrf2 also serves as a critical transcription factor in the development of fibrosis and antioxidant response. To be more specific, Nrf2 could dissociate from Keap1 and translocate to the nucleus. Subsequently, the transcriptional activity of Nrf2 is enhanced to activate the expression of some antioxidative genes [42, 43]. Previous study has unveiled that the activation of Nrf2 was also regulated by AKT, which was also involved in inflammatory and oxidative damage. Therefore, one of the limitations in the present study in that the status of Keap1/Nrf2 pathway was not investigated [44].

Natural therapeutic approaches have been recorded over 3,000 years. The secondary metabolites from natural products act as an endless frontier to develop compounds with medical and pharmaceutical purpose [45]. Green tea, a very popular beverage globally, is rich in flavonoids and phenolic acids, exhibiting important nutraceutical and medical properties [46]. EG is one of the most important polyphenolic compounds, owing seven hydroxyl radicals among three aromatic rings [47]. Previous studies reported that EG exerted antifibrotic effects by reducing collagen synthesis and blocking the major fibrosis-related pathways in keloids [48]. Also, in nonalcoholic fatty liver disease and cardiac remodeling, EG could decrease fibrogenic reaction and oxidative damage [18, 49]. Meanwhile, EG displays significant inhibitory effects on AKT/mTOR pathway in some diseases [50, 51]. In keeping with these studies, our results also disclosed that EG significantly decreased oxidative stress, apart from inhibiting the inflammatory factors including TNF-*α*, IL-1*β*, and MCP-1 in mice exposed to PM2.5. And the protective effects of EG on mice exposed to PM2.5 were mediated by AKT/mTOR pathway.

In conclusion, we found that EG improved PM2.5-induced lung fibrosis by decreasing oxidative damage and EMT in an AKT-dependent manner. The present study provides proofs for the application of EG in the treatment of PM2.5-induced lung fibrosis.

## Data Availability

All data supporting the findings in our study are available from the corresponding author upon reasonable request.

## References

[1] Aix M. L., Petit P., Bicout D. J. (2022). Air pollution and health impacts during the COVID-19 lockdowns in Grenoble, France. *Environmental Pollution*.

[2] Sun Q., Ren X., Sun Z., Duan J. (2021). The critical role of epigenetic mechanism in PM2.5-induced cardiovascular diseases. *Genes and Environment*.

[3] Guo X., Lin Y., Lin Y. (2022). PM2.5 induces pulmonary microvascular injury in COPD via METTL16-mediated m6A modification. *Environ Pollut*.

[4] Wang L., Cui Y., Liu H., Wu J., Li J., Liu X. (2022). PM2.5 aggravates airway inflammation in asthmatic mice: activating NF-*κ*B via MyD88 signaling pathway. *International Journal of Environmental Health Research*.

[5] McGuinn L. A., Ward-Caviness C. K., Neas L. M. (2016). Association between satellite-based estimates of long-term PM_2.5_ exposure and coronary artery disease. *Environmental Research*.

[6] Gao Y., Zhang Q., Sun J. (2022). Extracellular vesicles derived from PM2.5-exposed alveolar epithelial cells mediate endothelial adhesion and atherosclerosis in ApoE^−/−^ mice. *The FASEB Journal*.

[7] Dysart M. M., Galvis B. R., Russell A. G., Barker T. H. (2014). Environmental particulate (PM2.5) augments stiffness-induced alveolar epithelial cell mechanoactivation of transforming growth factor beta. *PLoS One*.

[8] Lv X., Liu C., Liu S. (2022). The cell cycle inhibitor P21 promotes the development of pulmonary fibrosis by suppressing lung alveolar regeneration. *Acta Pharmaceutica Sinica B*.

[9] Auyeung V. C., Downey M. S., Thamsen M. (2022). IRE1*α* drives lung epithelial progenitor dysfunction to establish a niche for pulmonary fibrosis. *American Journal of Physiology Lung Cellular and Molecular Physiology*.

[10] Roberts M. J., May L. T., Keen A. C. (2021). Inhibition of the proliferation of human lung fibroblasts by prostacyclin receptor agonists is linked to a sustained cAMP signal in the nucleus. *Frontiers in Pharmacology*.

[11] Duan J. X., Guan X. X., Yang H. H. (2021). Vasoactive intestinal peptide attenuates bleomycin-induced murine pulmonary fibrosis by inhibiting epithelial-mesenchymal transition: restoring autophagy in alveolar epithelial cells. *International Immunopharmacology*.

[12] Grigorieva O. A., Vigovskiy M. A., Dyachkova U. D. (2021). Mechanisms of endothelial-to-mesenchymal transition induction by extracellular matrix components in pulmonary fibrosis. *Bulletin of Experimental Biology and Medicine*.

[13] Ruan H., Gao S., Li S. (2021). Deglycosylated azithromycin attenuates bleomycin-induced pulmonary fibrosis via the TGF-*β*1 signaling pathway. *Molecules*.

[14] Lu Y., Zhang Y., Pan Z. (2022). Potential “therapeutic” effects of tocotrienol-rich fraction (TRF) and carotene “against” bleomycin-induced pulmonary fibrosis in rats via TGF-*β*/Smad, PI3K/Akt/mTOR and NF-*κ*B signaling pathways. *Nutrients*.

[15] Lin L., Zeng L., Liu A. (2021). Role of epigallocatechin gallate in glucose, lipid, and protein metabolism and L-theanine in the metabolism-regulatory effects of epigallocatechin gallate. *Nutrients*.

[16] Bulboacă A. E., Porfire A. S., Rus V., Nicula C. A., Bulboacă C. A., Bolboacă S. D. (2022). Protective effect of liposomal epigallocatechin-gallate in experimental gentamicin-induced hepatotoxicity. *Antioxidants*.

[17] Li B. Y., Li H. Y., Zhou D. D. (2021). Effects of different green tea extracts on chronic alcohol induced-fatty liver disease by ameliorating oxidative stress and inflammation in mice. *Oxidative Medicine and Cellular Longevity*.

[18] Cui Y., Wang Y., Liu G. (2021). Epigallocatechin gallate (EGCG) attenuates myocardial hypertrophy and fibrosis induced by transverse aortic constriction via inhibiting the Akt/mTOR pathway. *Pharmaceutical Biology*.

[19] Wang M., Zhong H., Zhang X. (2021). EGCG promotes PRKCA expression to alleviate LPS-induced acute lung injury and inflammatory response. *Scientific Reports*.

[20] Luo Z. L., Sun H. Y., Wu X. B., Cheng L., Ren J. D. (2021). Epigallocatechin-3-gallate attenuates acute pancreatitis induced lung injury by targeting mitochondrial reactive oxygen species triggered NLRP3 inflammasome activation. *Food & Function*.

[21] Shen H., Wu N., Liu Z., Zhao H., Zhao M. (2017). Epigallocatechin-3-gallate alleviates paraquat-induced acute lung injury and inhibits upregulation of toll-like receptors. *Life Sciences*.

[22] Zhao X. D., Liu H., Li T., Gong Q., Zhang W. L. (2017). Epigallocatechin Gallate attenuates hip fracture-induced acute lung injury by limiting mitochondrial DNA (mtDNA) release. *Medical Science Monitor*.

[23] Guohua F., Tieyuan Z., Xinping M., Juan X. (2021). Melatonin protects against PM2.5-induced lung injury by inhibiting ferroptosis of lung epithelial cells in a Nrf2-dependent manner. *Ecotoxicology and Environmental Safety*.

[24] Yang L., Liu G., Li X. (2020). Small GTPase RAB6 deficiency promotes alveolar progenitor cell renewal and attenuates PM2.5-induced lung injury and fibrosis. *Cell Death & Disease*.

[25] Hou L., Zhang J., Liu Y. (2021). MitoQ alleviates LPS-mediated acute lung injury through regulating Nrf2/Drp1 pathway. *Free Radical Biology & Medicine*.

[26] Ma Z. G., Yuan Y. P., Zhang X., Xu S. C., Wang S. S., Tang Q. Z. (2017). Piperine attenuates pathological cardiac fibrosis via PPAR-*γ*/AKT pathways. *eBioMedicine*.

[27] Zhang Y., Liu S., Li X., Ye J. (2021). Protective effect of fasudil on hydrogen peroxide-induced oxidative stress injury of H9C2 cardiomyocytes. *Disease Markers*.

[28] Xu M., Wang X., Xu L. (2021). Chronic lung inflammation and pulmonary fibrosis after multiple intranasal instillation of PM2.5 in mice. *Environmental Toxicology*.

[29] Zhang X., Liu Z., Yang W. (2022). Tetrahydrofolate alleviates the inhibitory effect of oxidative stress on neural stem cell proliferation through PTEN/Akt/mTOR pathway. *Oxidative Medicine and Cellular Longevity*.

[30] Zhang Y., Yin N., Sun A. (2020). Transient receptor potential channel 6 knockout ameliorates kidney fibrosis by inhibition of epithelial-mesenchymal transition. *Frontiers in Cell and Development Biology*.

[31] Saers J., Andersson L., Janson C., Sundh J. (2021). Respiratory symptoms, lung function, and fraction of exhaled nitric oxide before and after assignment in a desert environment--a cohort study. *Respiratory Medicine*.

[32] Tian D., Chen X., Hou P. (2022). Effects of exposure to fine particulate matter on the decline of lung function in rural areas in northwestern China. *Environmental Science and Pollution Research International*.

[33] Zhao T., Qi W., Yang P. (2021). Mechanisms of cardiovascular toxicity induced by PM_2.5_: a review. *Environmental Science and Pollution Research volume*.

[34] Zheng R., Tao L., Jian H. (2018). NLRP3 inflammasome activation and lung fibrosis caused by airborne fine particulate matter. *Ecotoxicology and Environmental Safety*.

[35] Li S., Cao S., Duan X. (2020). Long-term exposure to PM2.5 and children’s lung function: a dose-based association analysis. *Journal of Thoracic Disease*.

[36] Yang L., Lin Z., Wang Y. (2018). Nickle(II) ions exacerbate bleomycin-induced pulmonary inflammation and fibrosis by activating the ROS/Akt signaling pathway. *Environmental Science and Pollution Research International*.

[37] Wang J., Li Y., Zhao P. (2020). Exposure to air pollution exacerbates inflammation in rats with preexisting COPD. *Mediators of Inflammation*.

[38] Liu W., Gao C., Dai H. (2019). Immunological pathogenesis of membranous nephropathy: focus on PLA2R1 and its role. *Frontiers in Immunology*.

[39] Shen C., He Y., Chen Q. (2021). Narrative review of emerging roles for AKT-mTOR signaling in cancer radioimmunotherapy. *Ann Transl Med*.

[40] Wang J., Hu K., Cai X. (2022). Targeting PI3K/AKT signaling for treatment of idiopathic pulmonary fibrosis. *Acta Pharmaceutica Sinica B*.

[41] He Z., Chen S., Pan T. (2022). Ginsenoside Rg2 ameliorating CDAHFD-induced hepatic fibrosis by regulating AKT/mTOR-mediated autophagy. *Journal of Agricultural and Food Chemistry*.

[42] Li J., Yu Z., Han B. (2022). Activation of the GPX4/TLR4 signaling pathway participates in the alleviation of selenium yeast on deltamethrin-provoked cerebrum injury in quails. *Molecular Neurobiology*.

[43] Yang X., Fang Y., Hou J. (2022). The heart as a target for deltamethrin toxicity: inhibition of Nrf2/HO-1 pathway induces oxidative stress and results in inflammation and apoptosis. *Chemosphere*.

[44] Farooq U., Sahibzada M. U. K., Khan T. (2022). Folecitin isolated from hypericum oblongifolium exerts neuroprotection against lipopolysaccharide-induced neuronal synapse and memory dysfunction via p-AKT/Nrf-2/HO-1 signalling pathway. *Evidence-based Complementary and Alternative Medicine*.

[45] Condello M., Meschini S. (2021). Role of natural antioxidant products in colorectal cancer disease: a focus on a natural compound derived from prunus spinosa, trigno ecotype. *Cell*.

[46] Mancini E., Beglinger C., Drewe J., Zanchi D., Lang U. E., Borgwardt S. (2017). Green tea effects on cognition, mood and human brain function: a systematic review. *Phytomedicine*.

[47] Pervin M., Unno K., Takagaki A., Isemura M., Nakamura Y. (2019). Function of green tea catechins in the brain: epigallocatechin gallate and its metabolites. *International Journal of Molecular Sciences*.

[48] Park G., Yoon B. S., Moon J. H. (2008). Green tea polyphenol epigallocatechin-3-gallate suppresses collagen production and proliferation in keloid fibroblasts via inhibition of the STAT3-signaling pathway. *The Journal of Investigative Dermatology*.

[49] Tang G., Xu Y., Zhang C., Wang N., Li H., Feng Y. (2021). Green tea and epigallocatechin gallate (EGCG) for the management of nonalcoholic fatty liver diseases (NAFLD): insights into the role of oxidative stress and antioxidant mechanism. *Antioxidants*.

[50] Liao Z. H., Zhu H. Q., Chen Y. Y. (2020). The epigallocatechin gallate derivative Y_6_ inhibits human hepatocellular carcinoma by inhibiting angiogenesis in MAPK/ERK1/2 and PI3K/AKT/ HIF-1*α*/VEGF dependent pathways. *Journal of Ethnopharmacology*.

[51] Wei R., Penso N. E. C., Hackman R. M., Wang Y., Mackenzie G. G. (2019). Epigallocatechin-3-gallate (EGCG) suppresses pancreatic cancer cell growth, invasion, and migration partly through the inhibition of Akt pathway and epithelial-mesenchymal transition: enhanced efficacy when combined with gemcitabine. *Nutrients*.

